# Exercise Stress Echocardiography in the Diagnostic Evaluation of Heart Failure with Preserved Ejection Fraction

**DOI:** 10.3390/jcdd9030087

**Published:** 2022-03-17

**Authors:** Tomonari Harada, Kazuki Kagami, Toshimitsu Kato, Hideki Ishii, Masaru Obokata

**Affiliations:** 1 Department of Cardiovascular Medicine, Gunma University Graduate School of Medicine, Maebashi 371-8511, Gunma, Japan; tharada@gunma-u.ac.jp (T.H.); mirror.1028k@gmail.com (K.K.); t-kato@gunma-u.ac.jp (T.K.); hkishii@med.nagoya-u.ac.jp (H.I.); 2 Division of Cardiovascular Medicine, National Defense Medical College, Tokorozawa 359-8513, Saitama, Japan

**Keywords:** diagnosis, expired gas analysis, heart failure with preserved ejection fraction

## Abstract

More than half of patients with heart failure have a preserved ejection fraction (HFpEF). The prevalence of HFpEF has been increasing worldwide and is expected to increase further, making it an important health-care problem. The diagnosis of HFpEF is straightforward in the presence of obvious objective signs of congestion; however, it is challenging in patients presenting with a low degree of congestion because abnormal elevation in intracardiac pressures may occur only during physiological stress conditions, such as during exercise. On the basis of this hemodynamic background, current consensus guidelines have emphasized the importance of exercise stress testing to reveal abnormalities during exercise, and exercise stress echocardiography (i.e., diastolic stress echocardiography) may be used as an initial diagnostic approach to HFpEF owing to its noninvasive nature and wide availability. However, evidence supporting the use of this method remains limited and many knowledge gaps exist with respect to diastolic stress echocardiography. This review summarizes the current understanding of the use of diastolic stress echocardiography in the diagnostic evaluation of HFpEF and discusses its strengths and limitations to encourage future studies on this subject.

## 1. Introduction

The burden of heart failure (HF) is increasing worldwide, and more than half of patients with HF have a preserved left ventricular (LV) ejection fraction (HFpEF) [1]. Owing to aging populations and the increasing prevalence of metabolic comorbidities, such as obesity, metabolic syndrome, and diabetes mellitus, the prevalence and incidence of HFpEF relative to HF with reduced ejection fraction (HFrEF) is expected to increase further [2]. The limited treatment options for HFpEF and the increasing burden of this disease result in a substantial unmet clinical need in the modern era.

In contrast to HFrEF, the diagnosis of HFpEF is challenging because patients have a normal ejection fraction. In addition, many patients have normal hemodynamics at rest and show abnormal hemodynamic responses only during exercise [1,3]. Accumulating evidence has demonstrated the utility of exercise stress testing (exercise stress echocardiography or invasive hemodynamic exercise testing) in revealing diastolic reserve limitation and, subsequently, elevation in LV filling pressures during exercise. As a result, exercise stress testing has been increasingly recommended for the diagnostic evaluation of HFpEF [4,5,6,7,8,9]. For this purpose, exercise stress echocardiography (i.e., diastolic stress echocardiography) plays a central role in clinical practice owing to its noninvasive nature and wide availability [4]. However, evidence supporting the use of this method remains limited and many knowledge gaps remain. In this review, we will summarize the current understanding of the use of diastolic stress echocardiography for the diagnosis of HFpEF, highlighting its strengths and limitations.

## 2. Pathophysiological Background Supporting the Importance of Exercise Stress Echocardiography for the Diagnosis of HFpEF

The diagnosis of HFrEF is straightforward and requires the detection of a decreased ejection fraction on echocardiography in patients with symptoms of HF (e.g., dyspnea, peripheral edema, and jugular venous distention). In contrast, HFpEF is more difficult to evaluate because the patients’ LV ejection fraction is preserved, making it difficult to distinguish HFpEF from other disorders presenting with HF-like symptoms (e.g., pulmonary diseases) [3,10,11]. In such cases, the diagnosis relies on objective evidence of congestion or elevated LV filling pressures [10], including the detection of pulmonary congestion on chest radiography or computed tomography, high natriuretic peptide levels, echocardiographic signs of LV diastolic dysfunction, or abnormal central hemodynamics directly measured through cardiac catheterization. Nevertheless, the diagnosis of HFpEF is not simple because many patients, especially those with no or a low degree of congestion, have normal LV filling pressures at rest [4,12]. Thus, the methods for identifying HFpEF among euvolemic patients have poor sensitivity (~60%), even during right heart catheterization, when performed at rest [4]. In patients with HFpEF, intracardiac pressures dramatically increase during the stress of exercise [4,13,14,15,16,17]. On the basis of this hemodynamic background, the ability of exercise stress echocardiography to reveal abnormalities that develop only during stress has been receiving increasing attention [4,8,18,19].

## 3. Exercise Echocardiography Methods

### 3.1. Clinical Indications

Diastolic stress echocardiography is indicated in patients who are suspected of having HFpEF based on clinical history, symptoms, or physical findings but had no clear evidence of elevated filling pressures from assessments performed at rest, such as echocardiography or measurement of natriuretic peptide levels [3,4,10,20]. Patients with apparent congestion or abnormal findings indicative of elevated LV filling pressures (e.g., high left atrial pressure according to the European Association of Cardiovascular Imaging [EACVI]/American Society of Echocardiography [ASE] algorithm) do not need to be referred for exercise testing because of a sufficiently high pretest probability. Meanwhile, diastolic stress echocardiography should not be performed in patients with no features of HFpEF because even a positive exercise test does not increase the post-test probability of definitively diagnosing HFpEF (as, theoretically, the positive predictive value depends on disease prevalence) [21]. Therefore, diastolic stress echocardiography is most useful in patients with an intermediate pretest probability for HFpEF.

Two scoring systems are available for estimating the pretest probability of HFpEF: the H_2_FPEF and HFA-PEFF scores [8,22]. The H_2_FPEF score is a weighted composite score of four clinical factors (obesity, two or more antihypertensive medications, atrial fibrillation, and age > 60 years) and two echocardiographic parameters (ratio of early diastolic mitral inflow velocity to mitral annular tissue velocity [E/e′ ratio] > 9 and estimated pulmonary artery systolic pressure > 35 mmHg), ranging from 0 to 9 points [22]. The HFA-PEFF score is a consensus-based algorithm proposed by the Heart Failure Association (HFA) of the European Society of Cardiology (ESC) that is composed of three domains (functional, morphological, and natriuretic peptide domains) and ranges from 0 to 6 points [8]. Patients with an intermediate pretest probability based on these metrics (H_2_FPEF score of 2–5 points or HFA-PEFF score of 2–4 points) are indicated for diastolic stress echocardiography to refine the diagnosis of HFpEF [23]. Studies have also demonstrated that both scores predict reduced exercise capacity and clinical outcomes in patients with HFpEF [19,24,25,26,27].

### 3.2. Exercise Stress Methods

Exercise requires integrated physiologic responses in the cardiovascular system, including biventricular contractility, lusitropic effect, chronotropic response, systemic and pulmonary vasodilation, and venous return, involving the central and peripheral nervous systems, lungs, coronary circulation, and skeletal muscle [16,28,29,30]. Patients with HFpEF may develop abnormalities in several of these components, leading to symptoms of dyspnea and exercise intolerance [15,16,17,31]. When a patient is able to exercise, dynamic exercise (e.g., treadmill or bicycle ergometer testing) should be selected because it can cause physiologic stress to the cardiovascular system [7]. Isometric exercise (sustained isometric handgrip exercise) can be performed in some patients to produce afterload-mediated stress [32,33,34]. A preload stress test through passive leg raises or the leg-positive pressure maneuver might also represent a non-exercise test to reveal LV diastolic dysfunction [35,36]. However, multiple abnormalities contribute to LV diastolic dysfunction and elevated LV filling pressures in HFpEF [15,16,17,31]. Therefore, the use of handgrip exercises or a preload stress test is less likely to be an alternative to dynamic exercise because it only partially stresses the cardiovascular system (i.e., handgrip exercise does not considerably affect the heart rate, preload, or venous return) [5,37].

Supine bicycle stress echocardiography is the recommended modality for diastolic stress echocardiography, as it has important advantages over treadmill exercise, as follows [7,8,38]: it allows continuous image acquisition throughout the test rather than only immediately post-exercise; it uses the semi-left lateral decubitus position, which facilitates the acquisition of images during exercise; and it has a much lower risk of falling down than treadmill exercise. However, as most physical activities in daily living are performed in an upright position, upright bicycle ergometer exercise is likely to produce more physiological stress than supine bicycle exercise if diagnostic-quality images can be obtained during exercise [39,40]. Notably, because the exercise position can affect central hemodynamics, the results must be interpreted with caution [41]. An increase in LV filling pressures may be more prominent owing to greater preload augmentation in the supine position than in the upright position [4,39,40,42].

### 3.3. Stress Protocols, Image Acquisition, and Targeted Parameters

**Figure 1:**
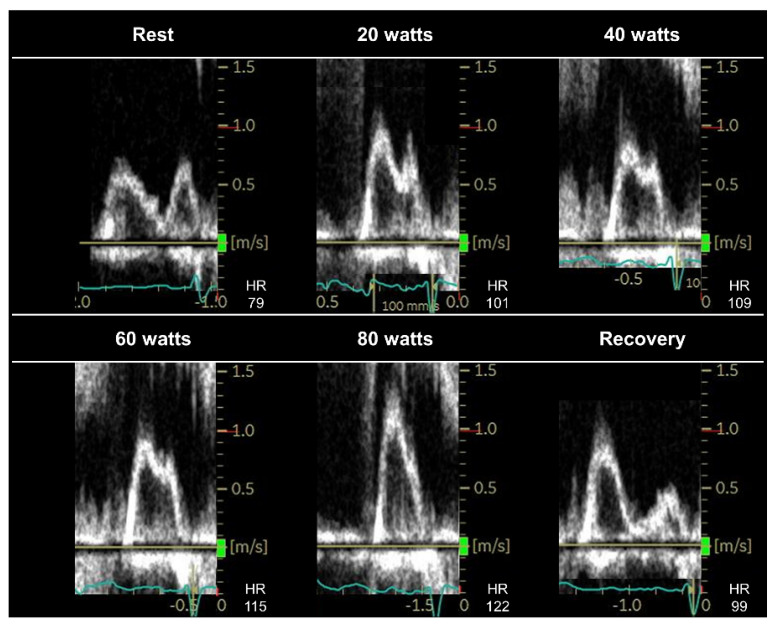
Changes in the transmitral inflow profile at rest and throughout exercise in a patient with heart failure and preserved ejection fraction. During peak exercise (80 watts), the transmitral E and A waves were indistinguishable owing to fusion. Continuous image acquisition allowed for the identification of an E wave of 100 cm/s at 60–watt exercise, and the E/e′ ratio at this stage was elevated (E/e′ ratio, 15.5). Invasive exercise right heart catheterization revealed that the pulmonary capillary wedge pressure (PCWP) was normal at rest (9 mmHg); however, it increased to 26 mmHg during peak exercise (80 watts). Although the E and A waves were no longer fused in the early recovery phase, the E/e′ ratio was 12.9. The invasively measured PCWP decreased to 19 mmHg (<25 mmHg) at 1 min post–exercise. HR, heart rate.

No universally adopted protocols exist for diastolic stress echocardiography. The EACVI/ASE guidelines recommend a multistage protocol, starting at 25 watts (W) at a 60 rpm cadence with the load increasing by 25 W every 3 min until peak exercise [7,20]. Other researchers have proposed a ramp stress protocol, starting at 15 W with 5 W increments every minute to a submaximal target heart rate of 100–110 bpm. During supine bicycle exercise echocardiography, echocardiographic images should be obtained at baseline, at each stage of exercise, and during the early recovery phase [7]. The advantage of image acquisition is the chance to determine the E/e′ ratio before the fusion of the mitral E and A velocities (Figure 1).

The EACVI/ASE guidelines recommend measuring the transmitral flow velocities, mitral annular tissue velocities, and tricuspid regurgitant velocity (TRV) to detect abnormal LV diastolic function reserve and the resulting increase in LV filling pressures [7,20]. The E/e′ ratio plays a key role in estimating LV filling pressures during exercise stress echocardiography [7,20]. A simultaneous catheterization–echocardiography study demonstrated a moderate correlation between the E/e′ ratio and invasively measured pulmonary capillary wedge pressure (PCWP) during exercise (r = 0.54–0.58) [4], although some studies raised questions about the ability of the E/e′ ratio to indicate changes in LV filling pressures [43,44,45]. The most common diagnostic limitation is the inability to evaluate the E/e′ ratio when the diastolic velocities are fused during high heart rates [4]. The EACVI/ASE guidelines recommend the acquisition of images during the submaximal phase before the fusion of the mitral E and A velocities (heart rate, 100–110 bpm) or during the early recovery period [7]. Previous studies using exercise right heart catheterization have consistently shown that an abnormal increase in left-side filling pressure occurs early during submaximal (20-W) exercise in patients with HFpEF [4,5,16,46,47]. This observation may support the utility of E/e′ ratio measurement during submaximal supine exercise in diagnosing HFpEF [4]; however, further studies are required to determine the diagnostic value of the E/e′ ratio during submaximal exercise, ideally using simultaneous invasive exercise hemodynamic testing. Conversely, PCWP may rapidly return to baseline levels early in the recovery phase even in patients with HFpEF (1 min post-exercise) [5]. Thus, PCWP may be normal when the E and A waves are no longer fused (Figure 1). It is also important to remember that E/e′ ratio cannot be applied to patients with specific diseases, such as mitral stenosis, severe mitral regurgitation, mitral annular calcification, mitral valve repair, or a prosthetic mitral valve, or in the presence of regional wall motion abnormalities [48].

Pulmonary hypertension (PH) in HF is primarily caused by a passive elevation in downstream LV filling pressures [49]. Thus, the assessment of PH during diastolic stress echocardiography is important to evaluate the severity of increases in LV filling pressures during the stress of exercise, and this can be achieved by measuring the TRV [7,8,20]. An isolated increase in the TRV during exercise is insufficient to diagnose HFpEF because it may be secondary to pulmonary vascular disease (pre-capillary component) or high cardiac output (CO). The presence of a concomitant increase in the TRV and E/e′ ratio can increase the probability of elevated LV filling pressures [8,50,51]. Importantly, exercise-induced PH predicts poor clinical outcomes in patients with HFpEF [52,53]. The current practice in assessing PH during exercise relies on TRV alone rather than the combination of TRV and right atrial pressure (RAP) [7,8,20,54]. This may be related to the technical difficulty in imaging the inferior vena cava during exercise; however, the exclusion of RAP leads to a serious underestimation of the severity of PH during exercise in some patients in whom RAP may increase dramatically (~40 mmHg) [14,15,17,55]. Prior studies reported a close relationship between RAP and peripheral venous pressure at rest [56,57]. Our group recently demonstrated that the measurement of peripheral venous pressure may be a reliable alternative to RAP measurement during diastolic stress echocardiography [58].

**Figure 2:**
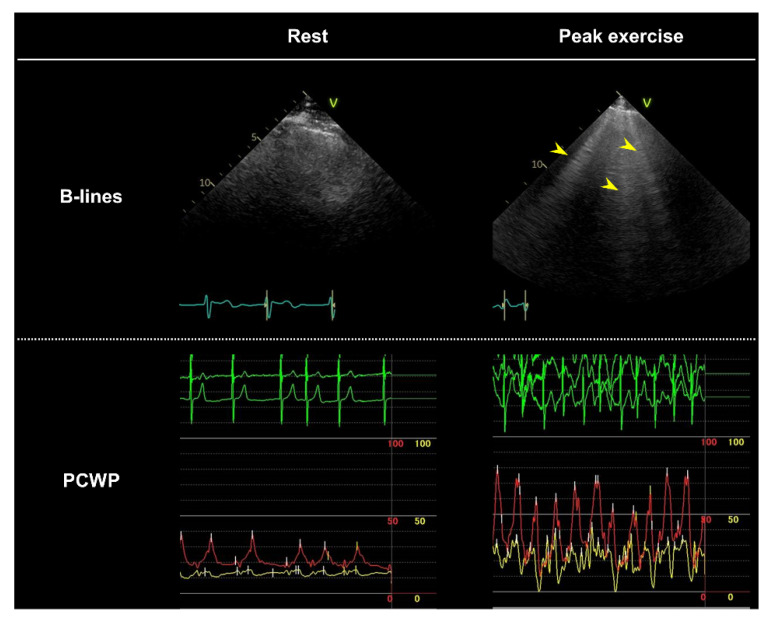
Exercise-induced ultrasound B-lines with simultaneously measured pulmonary capillary wedge pressure (PCWP) in a patient with heart failure and preserved ejection fraction. The patient demonstrated mildly elevated PCWP (19 mmHg, red line) without ultrasound B-lines at rest. During peak exercise (40 watts), the PCWP increased to 33 mmHg with marked V waves (71 mmHg), and multiple B-lines developed (yellow arrowheads).

Accumulating evidence has shown that lung ultrasound can reliably demonstrate pulmonary congestion as multiple B-lines in patients with HF [59]. Echocardiographic B-lines (lung comets) can be visualized as vertical, laser-like, hyperechoic artifacts that arise from the pleural line and extend to the bottom of the screen without fading [60,61]. B-lines may develop or quickly worsen in response to exercise in patients with HF, and “wet spots” may appear in the third intercostal space in two regions along the anterior axillary and mid-axillary lines, where B-lines most prominently develop during supine exercise [62]. An increased number of B-lines during exercise is correlated with hemodynamic congestion (Figure 2) (higher PCWP and pulmonary arterial pressures), as well as with reduced exercise capacity and worse clinical outcomes in HFpEF [63,64,65,66,67].

Notably, the assessment of ultrasound B-lines may be less influenced by the movement of the heart during exercise, contributing to a high data acquisition rate. However, the limitations in assessing B-lines in diastolic stress echocardiography also need to be considered. The presence of B-lines is not specific to pulmonary congestion but rather indicates interstitial syndrome [60,68]. Thus, its diagnostic value is limited in patients with concomitant interstitial lung diseases. B-lines can be evaluated in 28 chest regions [7]; however, scanning all 28 regions may reduce the time for imaging other parameters [63]. The most important limitation is the lack of consensus on the interpretation of results. Further studies are required to establish the cutoff value of the number of B-lines to define elevated LV filling pressures during exercise in patients with HFpEF.

**Figure 3:**
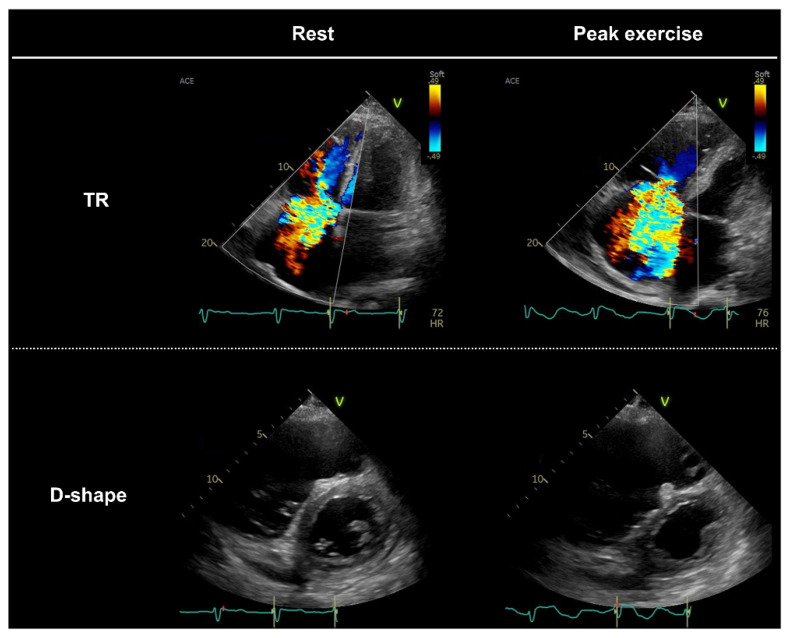
Heightened ventricular interdependence due to worsening tricuspid regurgitation (TR) during exercise in a patient with heart failure and preserved ejection fraction. The patient had persistent atrial fibrillation and moderate-to-severe TR at rest. The TR velocity was 2.5 m/s, and the estimated right atrial pressure based on inferior vena cava measurements was 15 mmHg. During peak exercise (20 watts), the TR dramatically worsened with incomplete coaptation of the tricuspid valves, resulting in paradoxical reduction in TR velocity (1.9 m/s). A significant increase in TR during exercise caused greater ventricular interdependence, contributing to reduced exercise capacity (peak oxygen consumption [VO_2_], 7.1 mL/min/kg).

Depending on the assumed differential diseases, additional echocardiographic parameters can be evaluated, including the following: regional wall motion; mitral regurgitation; pulmonary venous flow velocities; right ventricular systolic function (tricuspid annular plane systolic excursion, tricuspid lateral annular systolic velocity, or right ventricular longitudinal strain); tricuspid regurgitation; and inferior vena cava measurements. For example, regional wall motion abnormalities can be additionally evaluated in patients suspected of having HFpEF with multiple coronary risk factors. This approach might allow for a better pathophysiological characterization that may lead to a specific treatment strategy (Figure 3) [11].

### 3.4. Interpretation of Test Results and Diagnosis of HFpEF

**Figure 4:**
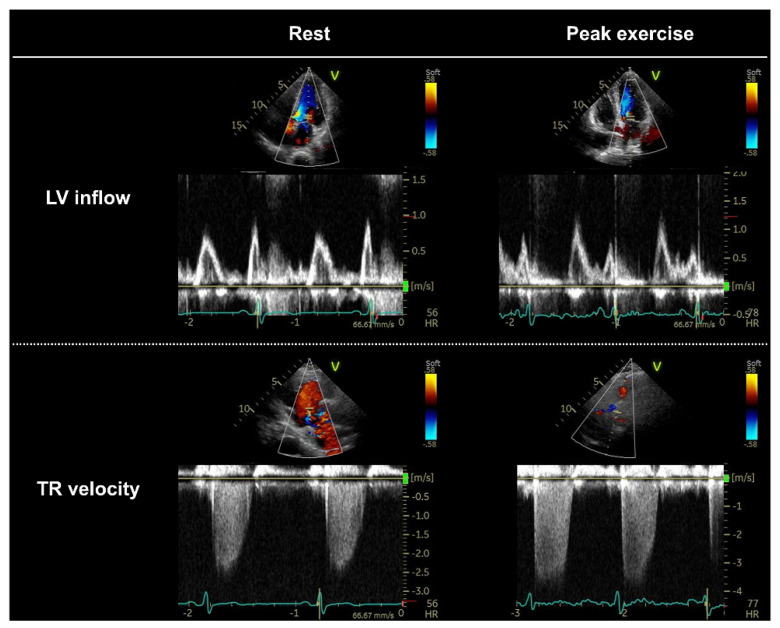
Key parameters in diastolic stress echocardiography. A 72-year-old woman who presented with exertional dyspnea was referred for diastolic stress echocardiography. She had a normal ejection fraction (61%), slightly elevated B-type natriuretic peptide (NP) levels (48.2 pg/mL), borderline E/e′ ratio (10.9), and a normal tricuspid regurgitation (TR) velocity (2.4 m/s). During peak exercise, the E wave and E/e′ ratio increased (16.9), with a concomitant elevation in TR velocity (3.8 m/s). LV, left ventricular.

**Figure 5:**
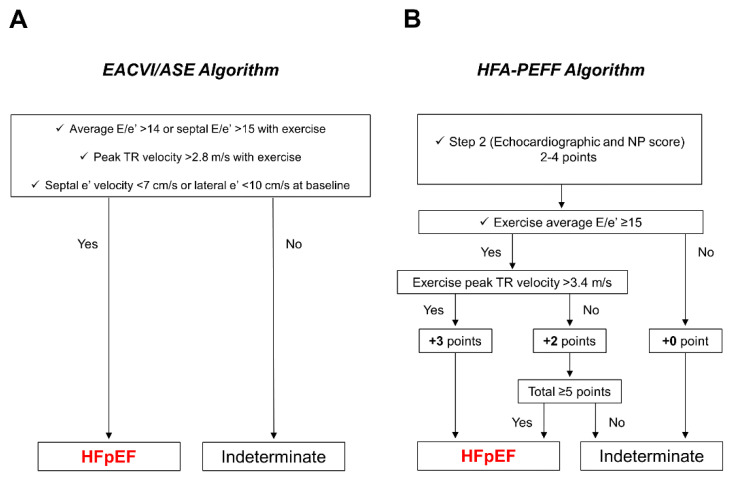
EACVI/ASE recommendations and HFA-PEFF algorithm for the diagnosis of HFpEF using exercise stress echocardiography. (**A**) In the EACVI/ASE recommendations, the test is considered abnormal (i.e., HFpEF) when all three criteria are met. (**B**) In the HFA-PEFF algorithm, the E/e′ ratio and TR velocity during exercise are used to add points to the resting HFA-PEFF score calculated in step 2. If the total score is ≥5 points, the diagnosis of HFpEF is confirmed. ASE, American Society of Echocardiography; EACVI, European Association of Cardiovascular Imaging; HFA-PEFF algorithm, a consensus-based algorithm proposed by the Heart Failure Association of the European Society of Cardiology; HFpEF, heart failure with preserved ejection fraction; TR, tricuspid regurgitation; NP, natriuretic peptide.

The EACVI/ASE proposed a consensus-based scheme to define abnormal diastolic function based on the E/e′ ratio, TRV, and e′ velocity (Figure 4 and Figure 5) [7,20].

Although this algorithm is pathophysiologically sound, its requirement of satisfying all three criteria may reduce the feasibility and sensitivity of diagnosing HFpEF. A study reported that during peak exercise, the E/e′ ratio was not measurable in approximately 20% and TRV was measurable in only approximately 50% of patients [4]. The HFA of the ESC suggested an algorithm that emphasizes the exercise E/e′ ratio, adding 2 points for isolated E/e′ elevation and 3 points for a concomitant increase in the E/e′ ratio and TRV to the resting HFA-PEFF score (Figure 5) [8]. This may be more probabilistically reasonable than the EACVI/ASE guidelines (i.e., patients with elevated E/e′ and TRV are more likely to have HFpEF). Thereby, the development or increasing number of B-lines may indicate an increased probability of HFpEF [3,63].

The most important limitation of exercise stress echocardiography is imaging quality. Acquiring diagnostic-quality images is more challenging during exercise in patients with obesity, which is very common in HFpEF. When echocardiographic imaging has poor quality or equivocal findings, invasive exercise hemodynamic testing is recommended to confirm the diagnosis [8].

## 4. Potential Value of Simultaneous Expired Gas Analysis

**Figure 6:**
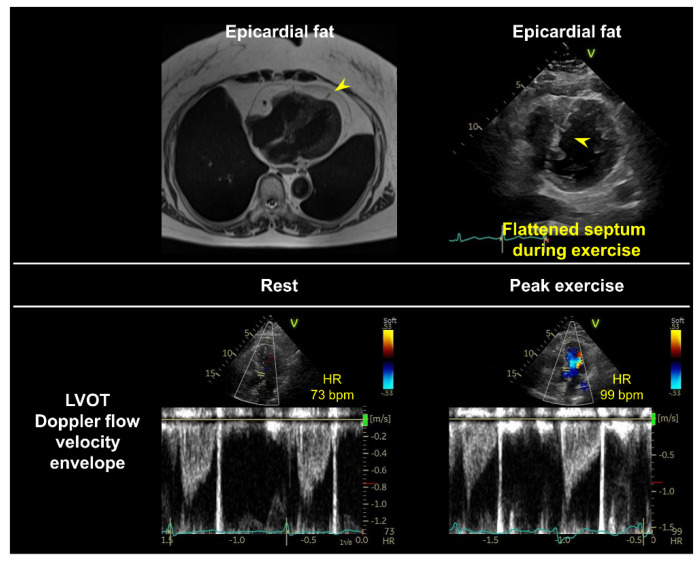
A case of cardiac output (CO) reserve limitation during exercise. A 79-year-old woman with obesity (body mass index, 30 kg/m^2^) was referred to our echocardiographic laboratory for the evaluation of unexplained dyspnea on exertion. Her NP levels were within the normal range (N-terminal pro-brain NP, 65 pg/mL). The results of resting echocardiography were also normal; however, cardiac magnetic resonance imaging showed remarkable epicardial fat tissue (yellow arrowhead). Diastolic stress echocardiography combined with expired gas analysis showed CO reserve limitation during exercise relative to increases in VO_2_ (CO, 2.9 to 3.9 L/min; VO_2_, 189 to 549 mL/min; CO reserve, 48%). The septum became flattened in the parasternal short-axis view during peak ergometry exercise (yellow arrowhead), suggesting that enhanced ventricular interdependence might have contributed to the CO reserve limitation due to reduction in LV preload in addition to chronotropic incompetence. LVOT, left ventricular outflow tract.

Cardiopulmonary exercise testing (CPET) is a gold standard test for evaluating exercise capacity and provides valuable information on exercise physiology involving the pulmonary, cardiovascular, and peripheral oxidative systems [69]. Notably, CPET-derived parameters are associated with clinical outcomes in both HFrEF and HFpEF [70,71,72]. Recently, interest has focused on combining diastolic stress echocardiography and expired gas analysis (i.e., CPET) in patients presenting with unexplained dyspnea and for the evaluation of HFpEF [16,39,50,66,67,69,73]. Peak oxygen consumption (VO_2_), especially percentage predicted value normalized to age, sex, and weight, is the gold standard objective marker of aerobic capacity in patients with cardiac dysfunction [69]. Previous studies have demonstrated that peak VO_2_ is universally decreased in patients with HFpEF, and expired gas analysis during exercise echocardiography enables the simultaneous assessment of reduced exercise capacity [14,15,16,40]. In this regard, very low or relatively preserved peak VO_2_ (<14 or >20 mL/min/kg) may be useful in identifying HFpEF among patients with dyspnea [39]. The factors limiting peak VO_2_ (or the O_2_ pathway) may vary among individual patients [14,15,16,30,40,74]. On the basis of the Fick principle (VO_2_ = CO × arteriovenous difference in O_2_ content), O_2_ delivery or convection and extraction are the two physiological determinants in the O_2_ pathway. Combining diastolic stress echocardiography with expired gas analysis also allows for the assessment of the CO reserve during exercise. CO can be estimated with echocardiography using the LV outflow pulse Doppler method. In healthy humans, a 6 mL/min increase in CO is required for a 1 mL/min increase in VO_2_ [75]. A CO reserve limitation is determined when the observed increase in CO is <80% of the predicted value based on the change in VO_2_ (Figure 6).

Peak VO_2_ measurement during diastolic stress echocardiography may also be useful for evaluating the therapeutic response. Prior studies have shown improvements in functional capacity after exercise training in patients with HFpEF by demonstrating changes in peak VO_2_ [76,77].

The ventilation equivalent to carbon dioxide production (V_E_/VCO_2_) slope represents ventilatory efficiency and is a strong prognostic marker in patients with HFrEF and HFpEF [70,78,79,80]. In patients with HF, ventilatory inefficiency, as evidenced by an increased V_E_/VCO_2_ slope, is likely to be a consequence of hemodynamic derangements during exercise or is a contributor to exercise intolerance [16,29,30,73]. Increased physiological dead space may be a primary contributor to impaired ventilatory efficiency in patients with HFpEF [29], and this could be associated with the presence of comorbid conditions including pulmonary vascular disease and chronic lung disease [16,81]. Further studies are warranted to determine the optimal use of combined exercise stress echocardiography and expired gas analysis for the diagnosis and evaluation of HFpEF.

## 5. Conclusions and Future Directions

Diastolic stress echocardiography plays an essential role in revealing abnormalities that develop only during exercise, and contemporary guidelines recommend its use as a diagnostic test to identify HFpEF among patients with unexplained dyspnea. Nevertheless, evidence supporting this practice remains limited and many unanswered questions and knowledge gaps remain (Table 1). Further studies are required to advance the knowledge of this method.

HFpEF is now understood as a heterogeneous syndrome, and phenotyping patients into pathophysiologically homogeneous groups may allow the personalization of new therapies [82]. Beyond resting assessments, diastolic stress echocardiography with simultaneous expired gas analysis may provide valuable information on the cardiac, pulmonary, and peripheral reserve during exercise and may improve understandings of the underlying pathophysiology and phenotypes of patients with HFpEF to facilitate better individualization of treatment.

## Figures and Tables

**Table 1:** Key Questions and Knowledge Gaps with Respect to Diastolic Stress Echocardiography.

Key Questions	Gaps in Evidence and Future Studies Needed
Diastolic stress echocardiography allows the diagnosis of HFpEF among patients with dyspnea; however, it is unclear whether early diagnosis itself will improve the clinical outcomes.	Echocardiographic markers of congestion during exercise are associated with clinical outcomes in HFpEF, supporting the prognostic value of diastolic stress echocardiography [53,65,83,84]; however, further prospective studies are needed to determine if intervention after an early diagnosis will improve the outcomes.
No universally adopted protocols exist, and whether a multistep or ramp protocol is better remains unknown.	Patients with HFpEF are older, and a ramp protocol or a multistep protocol with low initial and incremental workload (e.g., 10 watts) may be preferred [38]. Further studies are required to develop optimal protocols.
What is the optimal workload in identifying diastolic abnormalities? It is unclear whether maximal workload is necessary.	Submaximal exercise is likely to be more feasible and equivalent to daily activities; however, few studies have examined its diagnostic value [4]. Further studies are warranted to establish the clinical value of echocardiographic indices measured during submaximal exercise.
The E/e′ ratio plays a central role in diastolic stress echocardiography; however, what is the best way to address E–A fusion during exercise? What is the optimal cutoff of E/e′ during exercise in patients with AF? E/e’ ratio cannot be applied to patients with specific diseases, such as mitral valve diseases, mitral valve repair, or prosthetic mitral valves, or in the presence of regional wall motion abnormalities [52].	The E/e′ ratio during submaximal exercise or the early recovery period can be used to diagnose HFpEF; however, evidence supporting this practice is insufficient. Data on the exercise E/e′ ratio in patients with AF remain limited. Further studies are required to examine the diagnostic value of the exercise E/e′ ratio, using simultaneous invasive right heart catheterization.
Identification of pulmonary hypertension during exercise is useful for diagnosing HFpEF. Pulmonary hypertension may be underestimated in some patients, such as those with severe TR or those with very high RAP during exercise. How should this be addressed?	It is unclear how the underestimation of the TR gradient in patients with severe TR should be addressed. Further studies are required. Measurements of peripheral venous pressure may be a useful alternative to RAP measurements during exercise [56,58].
What is the diagnostic value of other candidate markers of congestion during diastolic stress echocardiography, such as echocardiographic B-lines or left atrial strain [85]?	The presence of multiple B-lines may be useful in detecting pulmonary congestion that develops during exercise [63]; however, it is unclear how the data should be interpreted (e.g., the optimal cutoff value for B-lines is unknown). Further studies are warranted to establish the optimal role of the assessment of B-lines in diastolic stress echocardiography.
What is the role of expired gas analysis combined with diastolic stress echocardiography?	Simultaneous assessment of exercise capacity (peak oxygen consumption) is the major advantage of diastolic stress echocardiography [39]. Further studies are needed to determine the clinical value of combining diastolic stress echocardiography and expired gas analysis in the diagnosis of HFpEF.

A, late diastolic mitral inflow velocity; AF, atrial fibrillation; E, early diastolic mitral inflow velocity; e′, early diastolic mitral annular tissue velocity; HFpEF, heart failure with preserved ejection fraction; RAP, right atrial pressure; TR, tricuspid regurgitation.

## Data Availability

Not applicable.
